# A method for real-time mechanical characterisation of microcapsules

**DOI:** 10.1007/s10237-023-01712-7

**Published:** 2023-03-24

**Authors:** Ziyu Guo, Tao Lin, Dalei Jing, Wen Wang, Yi Sui

**Affiliations:** School of Engineering and Material Science, Queen Mary University of London, London, E1 4NS United Kingdom

**Keywords:** Microcapsules, Real-time characterisation, Multilayer perceptron, Machine learning

## Abstract

Characterising the mechanical properties of flowing microcapsules is important from both fundamental and applied points of view. In the present study, we develop a novel multilayer perceptron (MLP)-based machine learning (ML) approach, for real-time simultaneous predictions of the membrane mechanical law type, shear and area-dilatation moduli of microcapsules, from their camera-recorded steady profiles in tube flow. By MLP, we mean a neural network where many perceptrons are organised into layers. A perceptron is a basic element that conducts input–output mapping operation. We test the performance of the present approach using both simulation and experimental data. We find that with a reasonably high prediction accuracy, our method can reach an unprecedented low prediction latency of less than 1 millisecond on a personal computer. That is the overall computational time, without using parallel computing, from a single experimental image to multiple capsule mechanical parameters. It is faster than a recently proposed convolutional neural network-based approach by two orders of magnitude, for it only deals with the one-dimensional capsule boundary instead of the entire two-dimensional capsule image. Our new approach may serve as the foundation of a promising tool for real-time mechanical characterisation and online active sorting of deformable microcapsules and biological cells in microfluidic devices.

## Introduction

A microcapsule is composed of a liquid droplet enclosed by a thin membrane that can resist shear deformation (Barthés-Biesel 2016). Microcapsules are commonly seen in nature and have been widely used as a mechanical model for living biological cells, such as the red blood cells (Pozrikidis 2003b; Freund 2014; Laumann et al. 2019) or circulating tumour cells (Takeishi et al. 2015; Xiao et al. 2017; Cui et al. 2021; Balogh et al. 2021). Artificial microcapsules have widespread pharmaceutical and biomedical applications, for instance, in drug delivery (Bhujbal et al. 2014) and encapsulated cell culture (Mayfield et al. 2014). The membrane mechanical properties of microcapsules, including the constitutive law and associated parameters such as the shear and area-dilatation moduli, determine the capsule’s mechanical strength and its response when resisting external forces. They are therefore important in the design and fabrication of microcapsules.

Determining the membrane mechanical properties of microcapsules is very challenging due to their tiny size and fragility. Conventional methods, such as the parallel plates compression (Carin et al. 2003), atomic force microscopy (Podskočová et al. 2005; Zhou et al. 2012), often apply a contacting force to the capsule, measure its response and fit the deformation to a theoretical prediction to inversely infer the capsule mechanical properties. Such methods often suffer from a low throughput rate, which is typically limited to 10-100 particles per hour (Wu et al. 2018).

It is also possible to deform the capsules with fluid flows, such as the linear shear (Chang and Olbricht 1993; Walter et al. 2001; deLoubens et al. 2016; Rahmat et al. 2019), centrifugal (Pieper et al. 1998; Husmann et al. 2005), extensional (deLoubens et al. 2014, 2015; Xie et al. 2017) and channel or tube flows (Lefebvre et al. 2008; Hu et al. 2013; Chu et al. 2013; Gubspun et al. 2016; Häner et al. 2020, 2021; Wang et al. 2021). In particular, extensional and channel/tube flows are flow-through platforms and therefore offer the potential for high-throughput measurement. Applied to biological cells, the state-of-the-art systems can classify tens to thousands of cells per second using microfluidic chips (Nitta et al. 2018; Isozaki et al. 2020; Saadat et al. 2020; Nawaz et al. 2020). However, mechanical properties of the cells often need to be calculated through post-processing of the experimental data, probably due to the long image processing and properties-prediction time of conventional inverse methods. Those methods often involve comparing the deformation of the bio-particle with a large number of samples obtained from theoretical predictions covering a large parametric space to identify the best fit.

In recent years, machine learning (ML) approaches, as powerful tools to infer the nonlinear relationship between data and their corresponding measurements, have attracted increasing attention from the biomechanical and biomedical communities. ML methods have been used in a wide range of applications including, for instance, cell morphological analysis (Phillip et al. 2021), cell classification (Nitta et al. 2018; Isozaki et al. 2020), disease diagnose (Jones et al. 2021; Peirlinck et al. 2019), patient-specific modelling (Saxby et al. 2020), blood flow reconstruction (Karniadakis et al. 2021) and control of fish swimming (Zhu et al. 2021). Lin et al. (2022) proposed a deep convolutional neural network (DCNN)-based prediction method which can identify the membrane constitutive law and estimate associated parameters of a microcapsule from its steady deformed profile in tube flow. Unlike conventional inverse methods which need to conduct a time-consuming process to identify the best fit during the prediction process, the DCNN-based approach is trained offline, and its prediction process only involves a limited number of algebraic calculations. It is therefore faster than conventional methods by several orders of magnitude and can predict the properties of more than one thousand capsules per second with high accuracy using graphics processing unit (GPU)-based parallel computing.

The DCNN-based approach of Lin et al. (2022, 2021a) calculates capsule mechanical properties from the two-dimensional (2D) binary image of the cross-sectional or footprint profile of the capsule in a tube or channel flow. From the feature maps of the DCNN, we find that the most relevant information is the profile of the capsule membrane, which is a one-dimensional (1D) curved boundary. The important observation indicates that the prediction efficiency may be improved significantly by developing ML algorithms that process the 1D capsule boundary only, instead of the entire 2D binary image that contains much unnecessary information. A short prediction time is crucially important for real-time online mechanical characterisation, or active mechanical property-based sorting. In the present study, we therefore propose a new approach, using multilayer perceptron (MLP), to drastically decrease the prediction time to less 1 millisecond per capsule using a personal computer. That is the total computational time, without using parallel computing, from a single experimental image to multiple capsule mechanical parameters. We test the performance of the present MLP-based method using both simulation and experimental data.

## Problem statement

**Fig. 1:**
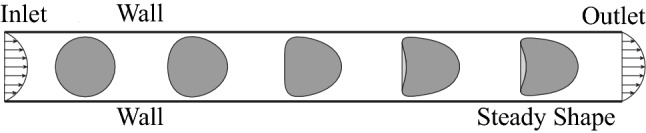
Illustration of an initially spherical capsule flowing in a capillary tube. The capsule reaches a steady shape after travelling a short distance. We use the footprint profile of the steady capsule to predict its membrane mechanical properties

We consider an initially spherical capsule of radius *a* flowing through a capillary tube with a radius *R* that is filled with a Newtonian fluid, as illustrated in Fig. 1. The capsule is enclosed by an infinitely thin hyper-elastic membrane, which can be described by one of the following three constitutive laws.

The strain-softening neo-Hookean (NH) law describes a volume-incompressible rubber-like isotropic material. For a 2D membrane, the area dilation is compensated by membrane thinning. The strain energy function of the 2D NH law follows:

1 $$\begin{aligned} W^{NH}=\frac{1}{2}G_s \left(I_1-1+\frac{1}{I_2-1} \right), \end{aligned}$$

where $W^{NH}$ is the strain energy density per unit undeformed surface area, $G_s$ is the membrane shear elasticity modulus, and $I_1$ and $I_2$ are the first and second strain invariants of the surface deformation. In the 2D NH law, the membrane area-dilatation modulus is related to the shear modulus by $K_s=3G_s$. The strain-hardening Skalak’s (SK) law (Skalak et al. 1973) was originally proposed to describe the membrane of red blood cells. Its strain energy function follows the form:

2 $$\begin{aligned} W^{SK}=\frac{1}{4}G_s (I_1^2+2I_1-2I_2)+\frac{1}{4}CG_s I_2^2, \end{aligned}$$

where *C* is a dimensionless parameter that measures the resistance to membrane area dilatation. It indicates the strength of the strain-hardening property of the membrane. In the SK law, the membrane area-dilatation modulus can be calculated from $K_s=(1+2C)G_s$. For biological membranes, *C* is usually much larger than unity, because of their quasi-area incompressibility. However, for bio-artificial capsules, the SK law has been found to fit experiments well when *C* is of order one (Rachik et al. 2006). Another membrane mechanical law that has been used for microcapsules with polymeric membranes is the 2D general Hooke’s law. It assumes linear elasticity and the strain energy function follows:

3 $$\begin{aligned} W^{H}=\frac{G_s}{4} \left(2I_1-2I_2+\frac{1}{1-\nu _s}I_1^2 \right), \end{aligned}$$

where $\nu _s$ is the surface Poisson’s ratio. In Hooke’s law, the membrane area-dilatation modulus is obtained from $K_s=(1+\nu _s)/(1-\nu _s)G_s$. A small bending rigidity, modelled with Helfrich’s formation (Zhong-can and Helfrich 1989; Cordasco and Bagchi 2013), has also been added to the present model to prevent membrane wrinkles. Therefore, the present model is mainly suitable for capsules with a thin membrane, where the effect of membrane bending on global capsule deformation is negligible (Dupont et al. 2015). We assume that the fluids inside and outside the capsule have identical density and dynamic viscosity. In fact, the viscosity ratio between the fluids inside and outside has no effects on the capsule’s steady deformation, because the internal fluid is largely in solid translation when the capsule has reached a steady shape.

We focus on the Stokes flow regime, where the capsule steady deformation is mainly determined by the following dimensionless parameters:

- the capillary numbers $Ca^{G_s}=\mu U/G_s$ and $Ca^{K_s}=\mu U/K_s$, which represent the ratios between viscous fluid force and membrane elastic forces due to shear and area dilatation, respectively;
- the confinement ratio $\beta =a/R$, which compares the size of the capsule to that of the tube;
- the pre-inflation ratio $\alpha =\frac{a}{a_0}-1$, which relates the initial inflated radius *a* to the radius $a_0$ of the capsule at unstressed configuration.

## Computational method

The present method consists of two parallel parts: the first part is a mechanistic approach, based on an immersed-boundary lattice Boltzmann method, to simulate the deformation of a capsule in tube flow in a wide parametric space. The simulation results, mainly the steady capsule profiles, together with the corresponding membrane constitutive laws and associated parameters, are then used to train an MLP-based neural network, which represents the second part of the present method. The trained MLP can be used to predict the membrane mechanical properties of microcapsules from their camera-recorded steady profiles in tube flow.

### Immersed-boundary lattice Boltzmann method

The immersed-boundary lattice Boltzmann method used in the present simulations has been extensively tested in previous studies of the deformation of capsules in shear or channel flows (Sui et al. 2008, 2008a, 2010; Wang et al. 2016, 2018; Lu et al. 2021; Lin et al. 2021). Details can be found in pertinent literature and here we only provide a brief overview. The flow field is modelled by a three-dimensional nineteen-speed (D3Q19) lattice Boltzmann model. At the wall of the tube, the no-slip boundary condition is implemented with a second-order interpolated bounce-back scheme (Bouzidi et al. 2001). A fully developed velocity profile at the channel inlet and outlet is imposed with a second-order non-equilibrium extrapolation scheme (Guo et al. 2002). The capsule is discrete into flat triangular elements. A finite element method is used to calculate the membrane elastic forces from the membrane deformation and its strain energy function. The interaction between the fluid and capsule membrane is solved by means of the immersed boundary method of Peskin (Peskin 1977).

### Multilayer perceptron

The MLP is one of the most widely used neural networks in supervised learning. It’s a fully-connected feed-forward network, which means a neuron of a layer is connected to all neurons of the next layer, and the information only flows from one layer to the next. Due to its simplicity and universal approximation property, the MLP has been widely used in problems such as design of airfoils (Sekar et al. 2019), and classification of cells (Chen et al. 2016; Nawaz et al. 2020).

#### Structure of the network

**Fig. 2:**
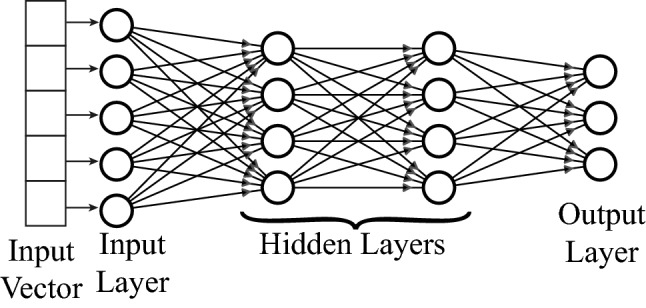
Illustration of the typical architecture of an MLP

The typical architecture of an MLP is illustrated in Fig. 2. It consists of input, hidden and output layers. Basic elements of the network are called neurons or perceptrons, which often conduct nonlinear input–output mapping defined by the regression problem. Each neuron receives weighted inputs from all neurons of the previous layer, and its output is calculated by passing the input signal through a nonlinear activation function. The output of the $j^{th}$ neuron in the $n^{th}$ layer can be obtained from:

4 $$\begin{aligned} x_j^n=\sigma \left (\sum _k w_{jk}^{n} x_k^{n-1}+b_j^n \right), \end{aligned}$$

where $w_{jk}^n$ is the weight of the connection from $k^{th}$ neuron of the $(n-1)^{th}$ layer to the $j^{th}$ neuron of the $n^{th}$ layer, and $b_j^n$ is the bias term. We have used the rectified linear unit (ReLU) as the non-linear activation function $\sigma (\cdot )$ due to its simplicity and superior ability to train the network faster (Goodfellow et al. 2016):

5 $$\begin{aligned} \sigma (x_j^n)=\text {max}(0,x_j^n). \end{aligned}$$

In the present method, the MLP predicts the capsule membrane mechanical law type and estimates the associated parameters, through classification and regression tasks, respectively, in the output layer. For classification tasks, we have used three neurons in the output layer, with the softmax function as the activation function (Eq. 6), to predict the probabilities that the capsule membrane belongs to the three law types:

6 $$\begin{aligned} \sigma (x_j)=e^{x_j}/\sum ^{3}_{j=1}e^{x_j}. \end{aligned}$$

We assume that the capsule membrane follows the constitutive law that has the highest probability.

#### Training

**Fig. 3:**
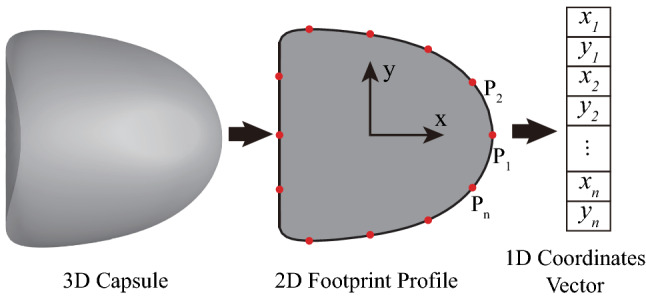
Illustration of the preparation of training samples of the MLP. The steady footprint profile of a capsule is discretised into equally spaced membrane nodes. The origin of the coordinate system is chosen as the mass centre of the capsule’s profile. Coordinates of the membrane nodes are built into a 1D vector, which serves as an input of the network

It should be noted that the samples for training of the present MLP have been obtained from numerical simulations using the immersed-boundary lattice Boltzmann method. A sample consists of a footprint profile of the steady shape of a capsule in tube flow, and its labels in the forms of the membrane constitutive law type and associated parameters. As illustrated in Fig. 3, we discretise the capsule profile into membrane nodes that have equal arc-length distance and assemble their coordinates into a 1D vector, which serves as an input of the present MLP.

**Fig. 4:**
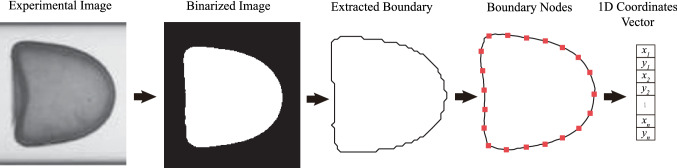
Illustration of the present image processing procedure which converts the experimental image of a capsule in tube flow into a 1D footprint-boundary coordinates vector that can be processed by the MLP

During training, internal parameters of the MLP, such as the weight and bias, are adjusted to minimise a loss function that describes how close the prediction is to the corresponding ground truth. In the present study, for regression tasks, we use the square loss function, which is defined as:

7 $$\begin{aligned} L^{reg}=\left| y^{label}-y\right| ^2, \end{aligned}$$

where *y* is the predicted value of a variable such as the $G_s$ or $K_s$, and $y^{label}$ is the corresponding ground truth. For classification of the membrane law type, we have used the cross-entropy loss function (Rubinstein 1999):

8 $$\begin{aligned} L^{cls}=-\sum _{j=1}^{3}z_j^{label}\text {log}(p_j) \end{aligned}$$

where $z_j^{label}$ is the ground truth probability that the capsule of the input image follows the $j^{th}$ type of membrane mechanical law, and $p_j$ represents the predicted probability.

To optimise the trainable parameters in the MLP, we obtain the gradients of the loss functions with respect to the trainable parameters and then update the values of the parameters with an optimiser based on a stochastic gradient descent algorithm called ADAM (Kingma and JL 2014). Mini-batch mode training is applied, and exposing all training samples to the MLP once is called an epoch. At the end of each epoch, the MLP is validated using a small portion ($10\%$ in the present study) of training samples that have not been actually used in training. With the process iterating, the losses decrease and converge to small values, indicating network predictions are approaching their corresponding ground truth. To avoid overfitting, batch-normalisation (Ioffe and Szegedy 2015) and dropout regularisation (Srivastava et al. 2014) have been applied. The training is terminated when the values of loss functions no longer decrease over several iterations even after reducing the learning rate.

The performance of the MLP is affected by its hyperparameters. In the present study, we conduct extensive tests to identify the optimum set of hyperparameters that minimises the values of the loss functions. The present MLP has three hidden layers with 128 neurons in each layer. During training, the mini-batch size is set as 128, and an initial learning rate of 0.001 has been used. In the dropout regularisation, the dropout rate is set as 0.3.

Once trained, the MLP can predict the membrane law type and estimate associated elastic parameters such as the $G_s$ and $K_s$ from the steady profile of a capsule in tube flow. In the present study, we first develop and train the MLP using the open-source framework Tensorflow v2.5 (Abadi et al. 2016). The framework is mostly based on Python, which runs codes through an interpreter, and is therefore not as efficient as compiled language such as the C++ (Langtangen 2008). To reduce computational time, we download the parameters of the trained MLP and reimplement the neural network using C++.

### Processing of experimental images

Note that the inputs of the present MLP consist of a 1D vector, which represents the coordinates of the membrane nodes that distribute evenly on the boundary of the footprint profile of a capsule (see Fig. 3), together with the corresponding labels. We choose to use the footprint profile instead of the cross-sectional profile, due to the fact that it becomes very challenging to obtain the latter using a fast algorithm when a capsule is undergoing large deformation where a part of its rear region hides in the shadow (see the capsule image in Fig. 4). In order to predict capsule properties from an experimental image, we have proposed an image processing procedure to convert the capsule image into a 1D coordinates vector that can be processed by the MLP.

The image processing procedure is illustrated in Fig. 4. Firstly, the camera-recorded image is subtracted with its background to remove the channel and static noise. The image is then binarised and the capsule is centred to a square region of interest (RoI) with a width of the tube diameter. Secondly, the boundary of the capsule in the binary image is detected with a border following algorithm proposed by Suzuki et al. (Suzuki and Abe 1985), and the boundary nodes are sorted counter-clockwisely. As can be seen in Fig. 4, the capsule boundary directly extracted from the binary image is not smooth. We find that it can significantly reduce the prediction accuracy of the present method. To solve this problem, a piecewise second-order polynomial function is employed to approximate the capsule boundary smoothly, and membrane nodes with equal arc-length distance are sampled from the fitted curve. Finally, coordinates of the membrane nodes are built into a 1D vector, in the same format of the training data.

## Results and discussion

### Tests with simulation results

We first test the accuracy of the present MLP by considering data generated from numerical simulations using the immersed-boundary lattice Boltzmann method. The ground truth information of the simulation results, such as the membrane law types and values of mechanical parameters, is known exactly. To train the MLP, we have used 400 sets of footprint profiles of capsules that have reached the steady state in tube flow. The capsule profile has been discretised into 60 equally spaced membrane nodes, as illustrated in Fig. 3. The training samples cover the NH, SK and Hooke’s laws, with $0.02\le Ca^{K_s}\le 0.055$ and $0.03\le Ca^{G_s}\le 0.21$. In this parametric space, high-quality experimental results of microcapsules with a polymeric membrane flowing in a glass tube are available, and the capsules can reach considerable deformation (Risso et al. 2006). The capsules have a confinement ratio of $\beta =0.77$. To mimic the swelling of microcapsules with a polymeric membrane in microfluidic flow due to an osmotic effect (Lefebvre and Barthes-Biesel 2007), the present capsules have been inflated slightly with a pre-inflation ratio of $\alpha =3\%$. Our testing samples are from the same parametric space and have not been used during the training of the MLP.

**Fig. 5:**
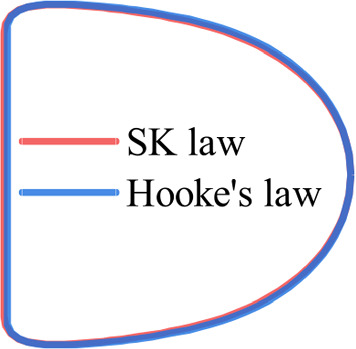
Comparison of the steady footprint profiles of capsules with different types of membranes (SK and Hooke’s, respectively) but the same $K_s$ and $G_s$. Capillary numbers are $Ca^{K_s}=$ 0.055, $Ca^{G_s}=0.165$

We find that the present MLP correctly predicts the membrane law type when the membrane of the capsule in a testing sample follows the NH law. However, the network cannot well discriminate the SK and Hooke’s laws. To solve the puzzle, we examine the steady profiles of capsules with the SK and Hooke’s membranes. When the capsule deformation is not too large, those capsules with the same membrane shear and area-dilatational elastic moduli $G_s$ and $K_s$ in the two mechanical laws have almost identical steady profiles. One example is shown in Fig. 5, for capsules with the two different types of membranes at $Ca^{K_s}=$ 0.055, $Ca^{G_s}=0.165$. Indeed for capsules with the SK and Hooke’s membranes under moderate deformation, it is the membrane elastic parameters instead of the law types that determine the capsule deformation in tube flow.

**Fig. 6:**
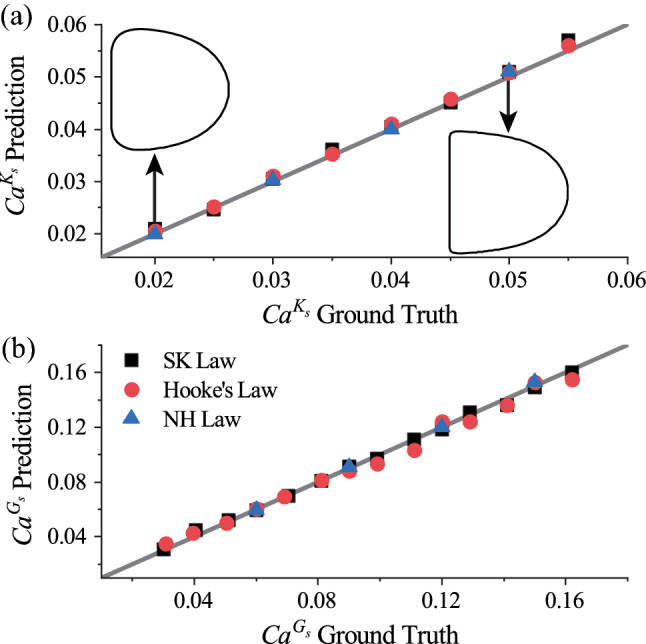
Comparisons of the predicted **a** $Ca^{K_s}$ and** b** $Ca^{G_s}$ with the corresponding ground truth. The solid lines are used as guides for the eyes representing perfect agreement. The insets in (**a**) are steady footprint profiles of capsules with an NH membrane which show the extent of capsule deformation. The membrane elastic moduli $K_s$ and $G_s$ are related to the capillary numbers by $K_s=\mu U/Ca^{K_s}$ and $G_s=\mu U/Ca^{G_s}$, respectively

We compare the MLP predicted $Ca^{K_s}$ and $Ca^{G_s}$ with the corresponding ground truth in Fig. 6 a & b respectively. Very good agreements can be observed. The insets in Fig. 6a are steady footprint profiles of capsules with an NH membrane at different $Ca^{K_s}$, which are used mainly to show the extent of capsule deformation. Note that the membrane elastic moduli $K_s$ and $G_s$ are related to the capillary numbers by $K_s=\mu U/Ca^{K_s}$ and $G_s=\mu U/Ca^{G_s}$, respectively. The mean absolute percentage errors (MAPEs) of the predicted $Ca^{K_s}$ and $Ca^{G_s}$ from the corresponding ground truth calculated from the entire testing samples are $4.2\%$ and $7.6\%$, respectively, which suggests excellent prediction accuracy. The MAPE of a parameter *y* is defined as:

9 $$\begin{aligned} MAPE(y)=\frac{1}{M}\sum _{i=1}^{M}\left| \frac{y_{true,i}-y_{predict,i}}{y_{true,i}}\right| , \end{aligned}$$

where *M* is the total number of the testing samples, $y_{predict,i}$ and $y_{true,i}$ are the predicted and ground truth values of the $i^{th}$ testing sample.

Note that it has been commonly believed that multiple experiments are necessary in order to measure the two membrane elastic moduli of a capsule $G_s$ and $K_s$ (Pozrikidis 2003a). The present results suggest that, with a neural network-based method, one may simultaneously infer both parameters with high accuracy from the steady profile of a capsule in tube flow, which can be obtained from a single experiment. We will further test our method in predictions of $G_s$ and $K_s$ from experimental images in the following Sect. 4.2.

Regarding the performance of a prediction method, besides accuracy, prediction time is another important parameter. A short prediction time is crucial for practical applications such as real-time characterisation of microcapsules, or active sorting of capsules based on their mechanical properties. For the results presented in Fig. 6, the time it has taken for the MLP to predict capsule membrane law type and estimate mechanical parameters averages 0.2ms, using a laptop computer with an AMD R7-4800U, 1.8GHz CPU. This is faster than a DCNN-based approach (Lin et al. 2022) that has similar accuracy with the same CPU by about two orders of magnitude. We will conduct a detailed comparison of the performance of the two types of neural networks in Sect. 4.2, where we predict capsule properties from experimental data.

**Fig. 7:**
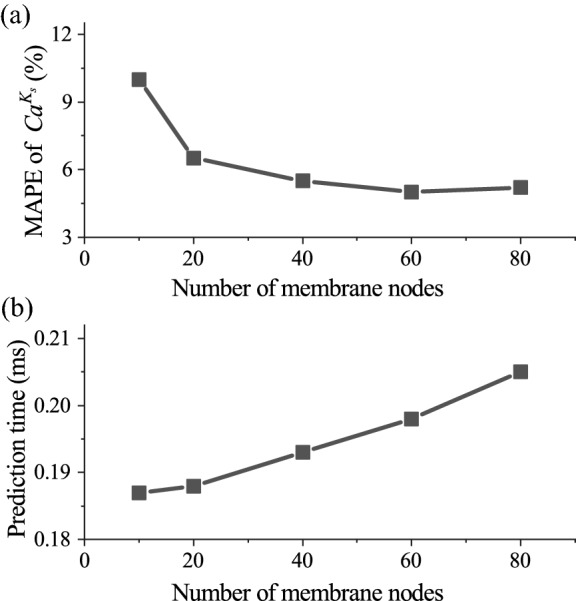
Effect of the number of membrane nodes representing a capsule’s steady footprint profile on **a** prediction accuracy, indicated by the MAPE of the predicted $Ca^{K_s}$ (the lower the MAPE, the higher the prediction accuracy), and **b** prediction time of the present MLP. The same training and testing data of Fig. 6 have been used

We consider the effect of the number of membrane nodes, which is, to some extent, equivalent to the resolution of the input of the MLP, on the prediction accuracy and time. The results are shown in Fig. 7. The prediction accuracy, indicated by the MAPE of the predicted $Ca^{K_s}$, generally increases with the number of membrane nodes. The MAPE reaches a plateau when there are 60 membrane nodes. It is also very encouraging to find from Fig. 7a that even with 10 membrane nodes, a reasonably low MAPE, i.e., $10.4\%$, can be reached. From Fig. 7b, it can be seen that with the number of membrane nodes increasing, the prediction time only increases slightly. This is due to the fact that we have used the same MLP in our tests of Fig. 7, where the number of hidden layers and neurons in each layer (except the input layer) remains identical.

### Tests against experimental results

Next, we use the same MLP model of Fig. 6 to predict the membrane constitutive law type and estimate the elastic moduli $G_s$ and $K_s$ of bio-artificial capsules with a human serum albumin-alginate membrane. The experiments were conducted by Risso et al. (2006), where capsules flowed through a capillary tube at different flow strengths. The capsules reached steady deformation and their profiles were taken by a camera. The capsule membrane area-dilatational modulus $K_s$ was measured with the parallel plate compression method (Risso and Carin 2004). The classical experimental data of Risso et al. (2006) have been widely used for bench-marking the accuracy of computational methods (Lefebvre and Barthes-Biesel 2007; Maestre et al. 2019; Lin et al. 2022).

#### Prediction accuracy

**Fig. 8:**
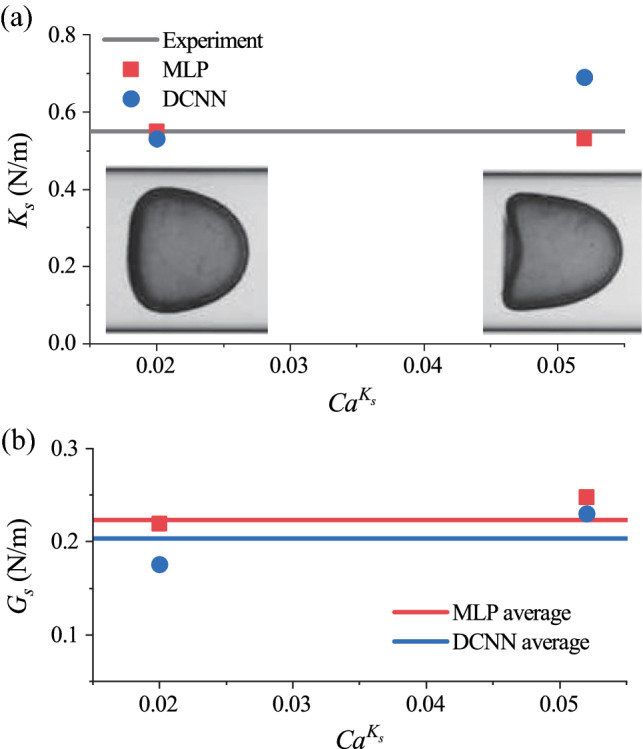
**a** Comparison of the predicted capsule membrane area-dilatational modulus $K_s$, using the MLP and DCNN, with that reported in experiments (Risso et al. 2006). The capsule was flowed through a tube at two speeds, leading to different values of $Ca^{K_s}$ and distinct levels of deformation. Photo insets are images of steady capsule profiles at the corresponding flow speed taken from Risso et al. (2006).** b** Predicted membrane shear modulus $G_s$ by the MLP and DCNN

Using the image processing procedure described in Sect. 3.3, we first covert the camera-recorded image of the steady profile of the capsule, shown in Fig. 8 where there are 138 pixels on the diameter of the undeformed capsule, into a 1D vector that consists of the coordinates of 60 equally distributed membrane nodes on the footprint profile of the capsule. The coordinates vector is then fed into the MLP which predicts the membrane law type and estimates values of the two elastic moduli $G_s$ and $K_s$. Note that the capsule images shown in Fig. 8 were taken from capsules with the same membrane properties flowing at different speeds, which has led to different values of $Ca^{K_s}$ and distinct levels of capsule deformation.

Our MLP has classified the capsule membrane as either SK or Hooke’s law, with comparable values of $G_s$ and $K_s$ associated with these two constitutive laws. The result is not surprising. As discussed in the previous Session 4.1, for capsules with SK or Hooke’s membrane undergoing moderate deformation in tube flow, it is the elastic parameters instead of the membrane law types that decide the capsule deformation. The MLP predicted membrane area-dilatational modulus $K_s$ is presented in Fig. 8a and is compared with the experimental measurement, where very good agreement can be observed. In the figure, we also present the predictions by a recently proposed DCNN-based approach (Lin et al. 2022), which deals with the 2D image of the capsule with the same image resolution. The accuracy of the present MLP seems to be better than that of the DCNN.

Note that in the experiments of Risso et al. (2006), the parallel plate compression method can only measure the membrane area-dilational modulus of the capsules. The membrane shear modulus $G_s$ of the capsules used in the experiments remains largely unknown. In early studies (Lefebvre and Barthes-Biesel 2007; Lin et al. 2022), researchers had assumed the value of *C* in the SK law to be unity to be able to infer the value of $G_s$ from the experiment images. The assumption may not be very accurate. The MLP and DCNN are both capable in simultaneous predictions of $G_s$ and $K_s$ from a single image of the steady profile of the capsule. The predictions of $G_s$ are shown in Fig. 8b. From the predicted values of $G_s$ and $K_s$ in Fig. 8, we find that the membrane area-dilatational parameter *C* in the SK law for the capsules of Risso et al. (2006) should be $C=0.73$, corresponding to a surface Poisson’s ratio of $\nu _s=0.42$ if using the 2D Hooke’s law.

#### Effect of image resolution

**Fig. 9:**
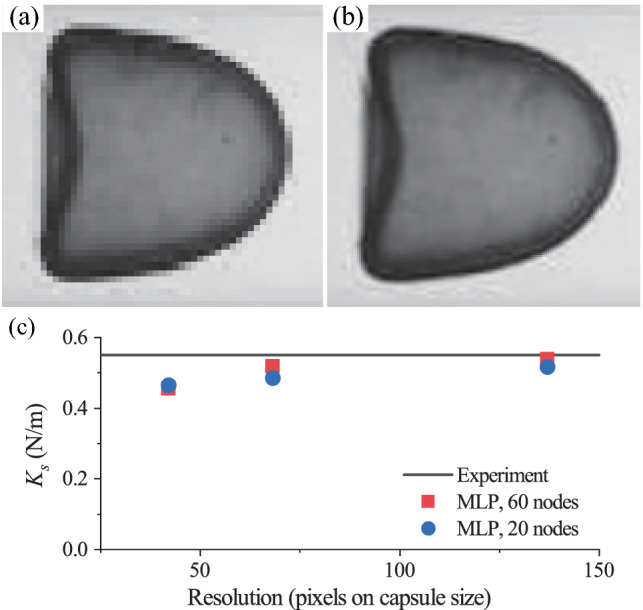
Effect of image resolution on prediction accuracy of the present MLP.** a**,** b** Capsules images with different resolutions. There are** a** 42 and** b** 138 pixels on the diameter of the undeformed capsule.** c** Comparison of predicted $K_s$, from images with different resolutions, with the experimental measurement (Risso et al. 2006)

We examine the effect of image resolution on the prediction accuracy of the present MLP. Figures 9a & b show two images of the same capsule at $Ca^{K_s}= 0.055$ with different resolutions: 42 and 138 pixels, respectively, on the diameter of the undeformed capsule. The capsule boundary in the low-resolution image shows a staircase shape and it becomes difficult for the method of Sect. 3.3 to accurately extract the boundary of the capsule.

We use the MLP to predict the $K_s$ of the capsules of Fig. 8 using images with three levels of resolutions and present the results in Fig. 9c. Note that in the tests the extracted capsule boundaries have all been discretised into 60 membrane nodes. From Fig. 9c, it is seen that the prediction accuracy increases with the image resolution. The relative error of the predicted $K_s$ from the experimental measurement decreases from $13\%$ to $2\%$, when the image resolution increases from 42 to 138 pixels on the capsule diameter.

Besides the image resolution, the number of membrane nodes used to represent the footprint profile of the capsule also affects the prediction accuracy. This has been demonstrated in the previous Sect. 4.1 where we have tested the present MLP using simulation results. A larger number of membrane nodes will enable a more accurate representation of the capsule profile, which generally leads to enhanced prediction accuracy. This is confirmed again in Fig. 9c where we also compare the prediction results of the MLP using capsules profiles represented by two levels of membrane nodes. Increasing the number of membrane nodes from 20 to 60, to better represent the capsule boundary that is extracted from the same image with a resolution of 138 pixels on the capsule diameter, will decrease the prediction error from 6% to 2%.

### Latency of prediction

**Table 1:** Image processing time, network prediction time and prediction error of the present MLP using sample images with different resolutions. The results are compared with the performance of a DCNN-based approach and an inverse method

	Image processing time (ms)	Prediction time (ms)	Prediction error (%)
MLP (42 pixels, 20 nodes)	0.25	0.19	12
MLP (42 pixels, 60 nodes)	0.47	0.2	13
MLP (68 pixels, 20 nodes)	0.32	0.19	11
MLP (68 pixels, 60 nodes)	0.54	0.2	6
MLP (138 pixels, 20 nodes)	0.45	0.19	6
MLP (138 pixels, 60 nodes)	0.76	0.2	2
DCNN (138 pixels)	0.44	54.2	14
Inverse method (138 pixels, 60 nodes)	0.76	18.7	2

We also examine the prediction latency of the present approach, which is defined as the time it takes for the algorithms to predict the capsule membrane law type and mechanical properties from a single camera-record image, without using any parallel computing. The prediction latency consists of two parts: the image processing time and network prediction time, both are affected significantly by the image resolution. The results, from the same tests of Fig. 9, are presented in Table 1. It is very encouraging to see that even with high accuracy, e.g., prediction error at 2%, the present MLP-base approach can reach a total latency of less than 1 ms. If we increase the tolerance to prediction accuracy and allow an error at the order of 10%, images with reduced resolutions can be used and the total prediction latency can drop to 0.44 ms.

In Table 1, we also compare the prediction latency of the present MLP-based approach with those of a recently proposed DCNN-based method (Lin et al. 2022, 2021a) and a Hausdorff distance-based inverse method. In the DCNN, image processing mainly involves binarisation of the 2D footprint profile of the capsule with a value of 1 inside the capsule and 0 outside. Then, the DCNN conducts convolution and pooling operations, using filters sliding through the entire 2D binary image. Since the DCNN deals with a 2D image instead of a 1D coordinates vector that has been used by the present MLP, and the convolution operations are computationally expensive, the latency of the DCNN is much longer than the MLP. In the present test, with the same AMD R7-4800U 1.8 G CPU, the DCNN prediction takes 54.2 ms, which is more than two orders of magnitude longer than the present MLP. We also find that the prediction time of the DCNN can be reduced to 11 ms when using a GPU (Tesla V100, 16GB, 1.38GHZ), that conducts parallel computing for convolution operations.

The inverse method infers the mechanical properties of a capsule from the best fit between experiments and computational predictions. Here, we compare the entire profile of a deformed capsule in experiment (i.e., $C_e$) with those obtained from numerical simulations $C_s$ (Mietke et al. 2015). The difference of two profiles is quantified with the mean Hausdorff distance (MHD) (Dubuisson and AK 1994). The minimum MHD indicates the best fit. To explain the MHD, let’s consider a set of *m* points, $R=\{r_1, r_2, r_3, \cdots r_m \}$ from $C_e$, and another set of *n* points, $T=\{t_1, t_2, t_3, \cdots t_n \}$ from $C_s$. Assuming that the two sets of points have the same centre of mass, the MHD ${\overline{h}}(R, T)$ is defined as:

10 $$\begin{aligned} {\overline{h}}(R, T)=\frac{1}{m}\sum _{r\in R} \min _{t\in T}[d(r, t)], \end{aligned}$$

where *d*(*r*, *t*) is the distance from any point in *R* to any point in *T*. From Table 1, we can find that with the same number of capsule boundary nodes, the image processing time in the inverse method is identical to that of the MLP, for images of the same resolution. The prediction accuracy of the two methods is also comparable. Regarding the prediction time, the MLP is about two orders of magnitude faster than the inverse method. That is due to the fact that the inverse method needs to scan all of the 400 data points to find the best fit, which is a time-consuming processing.

### Limitation and potential applications

A limitation of the present study is that our mechanical models only cover three classical membrane constitutive laws, which may not be able to predict the mechanics of some practical unknown capsules. The main purpose of this work is to demonstrate the real-time feature of the MLP-based method in mechanical characterisation of flowing microcapsules. In principle, our method can take into account more mechanical laws for different types of bio-microparticles including biological cells. However, one should note that the present methodology is not designed to create new mechanical laws from experimental data. For the latter purpose, some recently proposed data-driven approaches (Brunton et al. 2016) could be used.

The present approach may enable new applications in two important areas. The first is the real-time high-throughput mechanical characterisation of large population heterogeneous microcapsules or biological cells. Our approach lays down the software foundation for a practical system that can measure several millions of particles per hour. From the biomedical point of view, the system could make possible label-free screening of patient blood samples, which distinguishes healthy and diseased cells based on their mechanical properties, and use the information for diagnosis or drug efficacy monitoring. Secondly, our prediction method, due to its sub-millisecond computational latency, may lead to new technologies for label-free high-throughput active sorting of capsules or cells. In those technologies, mechanical properties are predicted at real time from deformation of particles flowing through a microfluidic channel and are used to classify cell/capsules types and trigger the downstream sorting module to pick up particles of specific types. Note that for processing biological cells, the present mechanical model will need to be upgraded. For particles undergoing small to moderate deformation, often the elastic parameters are better than the constitutive laws as indicators to distinguish particles. The constitutive laws may play more important roles when the particle deformation is very large.

## Conclusion

The main contribution of the present work is the development of a computational method, which combines a MLP-based ML approach with mechanistic capsule modelling, for real-time mechanical characterisation of flowing microcapsules. Our method can simultaneously predict the membrane law type and estimate multiple mechanical parameters from a single camera-recorded image of the steady profile of a capsule in tube flow. The accuracy of the present approach has been demonstrated through tests against both simulation and experimental results. With a reasonably high prediction accuracy and without using parallel computing, it takes less than 1 millisecond for the present MLP-based method to predict capsule mechanical properties from a single capsule image on a personal computer. This is faster than a recently proposed DCNN-based approach by two orders of magnitude, and it is due to the fact that the present MLP only processes the 1D boundary of the capsule, unlike the DCNN which conducts convolution operations over the entire 2D image. The present study therefore demonstrates the great potential of the MLP-based approach as an effective computational tool for real-time mechanical characterisation of microcapsules and, more broadly, deformable particles including biological cells.

## Data Availability

The codes for image processing, MLP prediction, and the testing data as well as a user’s guide can be found from the Github repository of the Queen Mary Biofluid Mechanics Laboratory: github.com/QMUL-Biofluids-Group/Real-Time-Mechanical-Characterisation-of-Microcapsules.
